# Correction: Using Assessment Design Decision Framework in understanding the impact of rapid transition to remote education on student assessment in health-related colleges: A qualitative study

**DOI:** 10.1371/journal.pone.0294196

**Published:** 2023-11-03

**Authors:** Myriam Jaam, Zachariah Nazar, Daniel C. Rainkie, Diana Alsayed Hassan, Farhat Naz Hussain, Salah Eldin Kassab, Abdelali Agouni

There are errors in the Funding section. The correct Funding statement is: Open Access funding provided by the Qatar National Library.
