# Supplementary material for: Using Assessment Design Decision Framework in understanding the impact of rapid transition to remote education on student assessment in health-related colleges: A qualitative study
Source: PLoS One. 2021 Jul 9;16(7):e0254444. doi: 10.1371/journal.pone.0254444 (PMC8270116; doi:10.1371/journal.pone.0254444)
Supplement: S1 Table — (DOCX) [file pone.0254444.s001.docx]

| **Table 1. Participants characteristics** | | | |
| --- | --- | --- | --- |
| **Code** | **Role** | **College/year** | **Professional Year / position** |
| SP1 | Student | Pharmacy | 4^th^ year |
| SP2 | Student | Pharmacy | 4^th^ year |
| SP3 | Student | Pharmacy | 4^th^ year |
| SP4 | Student | Pharmacy | 3^rd^ year |
| SP5 | Student | Pharmacy | 2^nd^ year |
| SP6 | Student | Pharmacy | 3^nd^ year |
| SP7 | Student | Pharmacy | 3^rd^ year |
| SP8 | Student | Pharmacy | 1^st^ year |
| SP9 | Student | Pharmacy | 2^nd^ year |
| SP10 | Student | Pharmacy | 1^st^ year |
| SP11 | Student | Pharmacy | 1^st^ year |
| SP12 | Student | Pharmacy | 2^nd^ year |
| SP13 | Student | Medicine | 4^th^ year |
| SP14 | Student | Medicine | 3^rd^ year |
| SP16 | Student | Medicine | 1^st^ year |
| SP17 | Student | Medicine | 1^st^ year |
| SP18 | Student | Pubic health | 3^rd^ year |
| SP19 | Student | Public health | 4^th^ year |
| SP20 | Student | Public health | 4^th^ year |
| FP1 | Faculty | Pharmacy | Associate professor |
| FP2 | Faculty | Pharmacy | Associate professor |
| FP3 | Faculty | Public Health | Associate professor |
| FP4 | Faculty | Public health | Associate professor |
| FP5 | Faculty | Public health | Assistant professor |
| FP6 | Faculty | Pharmacy | Lecturer |
| FP7 | Faculty | Medicine | Teaching Assistant |
| FP8 | Faculty | Medicine | Assistant professor |
| FP9 | Faculty | Pharmacy | Assistant professor |
| FP10 | Faculty | Medicine | Assistant professor |
| FP12 | Faculty | Public health | Lecturer |
| FP13 | Faculty | Medicine | Clinical Tutor |
| FP14 | Faculty | Pharmacy | Assistant Professor |
