# Supplementary material for: Using Assessment Design Decision Framework in understanding the impact of rapid transition to remote education on student assessment in health-related colleges: A qualitative study
Source: PLoS One. 2021 Jul 9;16(7):e0254444. doi: 10.1371/journal.pone.0254444 (PMC8270116; doi:10.1371/journal.pone.0254444)
Supplement: S1 File — (DOCX) [file pone.0254444.s002.docx]

# Supplementary File – Interview Guides.

# Faculty interview Guide

| **Start (recording)** |
| --- |
| **Welcome**: Interviewer will welcome the participant  **General introduction:** The interviewer will introduce him/herself.  **Example**  “Thank you for being here today. My name is ___, a research investigator at Qatar University. The main purpose of this interview is to understand your perspective about the assessments conducted during COVID19 pandemic. We are very interested to hear your opinions and learn from your experiences regarding the new assessments… The interview is a method of learning from experience both positive and negative. We are not trying to achieve consensus; we are just gathering information to help us have an in-depth understanding of the context…” |
| **Ground Rules** |
| 1. **We want you to do the talking. Talk freely; there is no right or wrong answers.** 2. **Try to reflect on your experiences with the new assessments** 3. **Whatever is mentioned within interview, will not be linked back to you** 4. **We will record the discussion to capture everything you have to say, but we will not identify anyone by name in our report, you will remain anonymous. This is to help us not to miss anything important that you say and so that we revisit the information during transcribing if necessary. Records will be deleted once transcribed.** 5. **You may refuse to answer any question or withdraw from the study at anytime** 6. **We would appreciate if you could please turn off your mobile phone to avoid distractions during the session.** 7. **The duration of this interview is about 45 – 60 minutes.** |
| **Participant’s introduction and demographics** |
| Ask participants  To introduce themselves (student or faculty)  To introduce their college |
| Faculty:  How long have you been teaching?  How long have you been in Qatar University? |

We are focusing on graded assessments conducted post COVID19 (this includes exams and assignments)

In answering the below questions think of a course you are coordinating in the undergraduate level.

| Purpose | - What new assessments (New to the course? New to the instructor (i.e. never been used)?) were used to capture the course learning objectives during the COVID19 period? - How did the assessment conducted post COVID19 support student learning?   Prompts:   - Was it more formative assessments or summative assessments? - Did the new assessments help students make judgements about their own learning? How so? - Did you feel that the students were focusing on grades or on the learning itself? |
| --- | --- |
| Context | How did you consider characteristics of enrolled students when designing assessment plan?  Prompts:   - What assumptions did you have about your learners (e.g. prior learning, skills such as technological proficiency)? How accurate do you think these are? - Are there identifiable subgroups of learners in your course? If so, what implications did this have for the assessment tasks and different learners’ needs for support? - How do you think your expectations and your learners’ expectations of assessment differ?   How did you consider QU assessment principles and policies when designing assessment plan?  Prompts:   - Were there any guidelines that helped you plan your assessment? How helpful were these? - What logistics and resources were required to support the assessment?   How did you consider learning environment, e.g. class size or mode (online/face-to-face/blended) when designing assessment plan?   - What are the benefits and limitations of the assessment environments for the educators? |
| Outcomes | How does the new assessment align with, and promote, desired course learning outcomes?  Prompts:   - Were there any outcomes you could not assess? - Were the new assessments harder/more complex than previously? - Were there any non-graded activities (in-class or online) that helped learners complete the assessment? - Were there any experiences which might have influenced how you designed the assessment? |
| Tasks | What was the rationale for each assessment?  What was the criteria for successful completion?  How were the assessments distributed across the remainder of the semester including their relationship with other assessments within the program?  Prompts:   - How did the design of the assessment discourage plagiarism? - What do you think about the use of cameras in exams? - Were there shortcuts students could use to bypass the learning but still complete the assessment? - Were rubrics developed, and if so, how? Was the rubric affected by prior student performance? - How much time did you expect each assessment to take learners? Were these reasonable estimates? - How were issues of validity and equity (such as ensuring the assessment is a fair indication of learners’ capacities or that assessors are suitably prepared) addressed? - How did you ensure that learners’ and assessors’ workloads are not overly burdensome? |
| Feedback | Did students have opportunities to engage in feedback? after the assessment?  How were the learner’s performance and feedback used to influence the (re)design of later assessments?  Prompts:   - When, in relation to the assessment, were the feedback given? - What were the most important matters that feedback focused on? |
| Interactions | How did resistance or engagement from students influence the assessment processes?  After changing the assessments, what associated changes in teaching and learning activities were required?  Prompts:   - Were the learners familiar with the kind of assessments you used? - were there adequate opportunities for learners to discuss and clarify what is expected? - What were the benefits and drawbacks of the new assessments? |
| Ending | If you were to go through the process again, what changes would you make to assessment process? |

# Students interview guide

| **Start** |
| --- |
| **Welcome**: Interviewer will welcome the participant  **General introduction:** The interviewer will introduce him/herself.  **Example**  “Thank you for being here today. My name is ___, a research investigator at Qatar University. The main purpose of this interview is to understand your perspective about the assessments conducted during COVID19 pandemic. We are very interested to hear your opinions and learn from your experiences regarding the new assessments… The interview is a method of learning from experience both positive and negative. We are not trying to achieve consensus; we are just gathering information to help us have an in-depth understanding of the context…” |
| **Ground Rules** |
| 1. **We want you to do the talking. Talk freely; there is no right or wrong answers.** 2. **Try to reflect on your experiences with the new assessments** 3. **Whatever is mentioned within interview, will not be linked back to you** 4. **We will record the discussion to capture everything you have to say, but we will not identify anyone by name in our report, you will remain anonymous. This is to help us not to miss anything important that you say and so that we revisit the information during transcribing if necessary. Records will be deleted once transcribed.** 5. **You may refuse to answer any question or withdraw from the study at anytime** 6. **We would appreciate if you could please turn off your mobile phone to avoid distractions during the session.** 7. **The duration of this interview is about 45 – 60 minutes.** |
| **Participant’s introduction** |
| Ask participants  To introduce themselves (student or faculty)  To introduce their college |

To clarify, assessments include exams, assignments and tasks which you were assessed on. We are focusing on the new/changed assessments during the COVID19 period.

| Purpose | - How did the assessment conducted post COVID19 support your learning?   Prompts:   - What new assessments were used to capture the course learning objectives? - Were you provided with rubrics? Did you feel that they reflect on the course objectives? - Did the new assessments help you make judgements about your own learning? How so? - Were you concerned about the grades? |
| --- | --- |
| Context | What expectations did you have about the assessments?  What kind of support did you need for completing the assessments?  Prompts:   - Were you provided with any guidelines on how to complete the assessments? - What logistics and resources were required to support the assessment? - What are the benefits and limitations of doing the assessment at home? |
| Outcomes | From your perspective did the new assessments capture the overall course learning outcomes?  Prompts:   - Were there any outcomes not assessed? - Were there any non-graded activities (in-class or online) and feedback that helped you complete the assessment? - Are there further skills and knowledge you feel you need to develop in that will help in your future professional life? - Were there any experiences which might have influenced how you performed in the assessment? |
| Tasks | Were you informed about the rationale for each assessment?  How were the assessments distributed across the remainder of the semester including their relationship with other assessments within the program?  Prompts:   - How did the design of the assessment discourage plagiarism? - Were there shortcuts students could use to bypass the learning but still complete the assessment? - What preparatory learning and teaching activities were necessary for you to commence these assessments? - What were your expectations for grading? - How much time did it take you to complete the assessment? Was the time provided by the instructor enough? |
| Feedback | Did you have opportunities to engage in feedback?  What types of feedback information were provided and by whom?  Prompts:   - When, in relation to the assessment, were the feedback given? - What form did the feedback take? - What were the most important matters that feedback focused on? |
| Interactions | What information were needed to improve subsequent assessments?  What associated changes in teaching and learning activities were required?  Prompts:   - What did you see as benefits regarding the new assessments? - What did you see as drawbacks or negative outcomes? - Were you familiar with the kind of assessments used? |
