# Supplementary material for: Using Assessment Design Decision Framework in understanding the impact of rapid transition to remote education on student assessment in health-related colleges: A qualitative study
Source: PLoS One. 2021 Jul 9;16(7):e0254444. doi: 10.1371/journal.pone.0254444 (PMC8270116; doi:10.1371/journal.pone.0254444)
Supplement: Attachment — Submitted filename: Response to Reviewers.docx [file pone.0254444.s003.docx]

May 9, 2021

The Editor-in-Chief

PLOS ONE

Dear Madam/Sir,

**Revision of Manuscript PONE-D-21-07338 Using Assessment Design Decision Framework in understanding the impact of Rapid Transition to Online-learning on Student Assessment in Health-related Colleges: A Qualitative Study**

The authors of the above-named manuscript would like to thank you for your e-mail dated 7 May 2021, containing the constructive comments of the reviewer. The authors very much appreciate the comments and have revised the manuscript accordingly as attached. We are positive that the comments helped in enhancing the quality and scientific merit of the paper.

Below are separate pages, detailing our responses to the comments of the journal requirements and reviewer.

**Please note that changes are made using “track changes” tool within the revised manuscript.**

Thank you to the editor and the reviewer for the time spent on this manuscript. We look forward to receiving your kind response.

Sincerely yours,

**The Corresponding Author**

**All in all, we thank you for your great review which we believe would add great value to the adjusted manuscript.**

**Detailed Responses to the Comments of the Journal Requirements and Reviewer**

| Journal Requirements | Author’s response |
| --- | --- |
| 1. Please ensure that your manuscript meets PLOS ONE's style requirements, including those for file naming. The PLOS ONE style templates can be found at  <https://journals.plos.org/plosone/s/file?id=wjVg/PLOSOne_formatting_sample_main_body.pdf> and  <https://journals.plos.org/plosone/s/file?id=ba62/PLOSOne_formatting_sample_title_authors_affiliations.pdf> | The manuscript was updated to comply with the style required |
| 2. We note that you have indicated that data from this study are available upon request. PLOS only allows data to be available upon request if there are legal or ethical restrictions on sharing data publicly. For information on unacceptable data access restrictions, please see <http://journals.plos.org/plosone/s/data-availability#loc-unacceptable-data-access-restrictions>  In your revised cover letter, please address the following prompts:  2a) If there are ethical or legal restrictions on sharing a de-identified data set, please explain them in detail (e.g., data contain potentially identifying or sensitive patient information) and who has imposed them (e.g., an ethics committee). Please also provide contact information for a data access committee, ethics committee, or other institutional body to which data requests may be sent.   2b) If there are no restrictions, please upload the minimal anonymized data set necessary to replicate your study findings as either Supporting Information files or to a stable, public repository and provide us with the relevant URLs, DOIs, or accession numbers. Please see <http://www.bmj.com/content/340/bmj.c181.long> for guidelines on how to de-identify and prepare clinical data for publication. For a list of acceptable repositories, please see <http://journals.plos.org/plosone/s/data-availability#loc-recommended-repositories>. | The following is also added in the cover letter as required.  “there are ethical restrictions on sharing these data since it contains potentially identifying participant information. These restrictions are imposed by Qatar University Institutional Review Board (QU-IRB). Therefore, for access to these data, the corresponding author should be contacted or the QU-IRB office through: QU-IRB@qu.edu.qa or through 0097 44403-5307 with approval reference number: 1283-EA/20” |
| 3. Please amend either the abstract on the online submission form (via Edit Submission) or the abstract in the manuscript so that they are identical. | The authors have checked and made sure the abstract in the system and within the manuscript is identical. |
| 4. Please review your reference list to ensure that it is complete and correct. If you have cited papers that have been retracted, please include the rationale for doing so in the manuscript text, or remove these references and replace them with relevant current references. Any changes to the reference list should be mentioned in the rebuttal letter that accompanies your revised manuscript. If you need to cite a retracted article, indicate the article’s retracted status in the References list and also include a citation and full reference for the retraction notice. | The authors reviewed each reference and all references were updated. Additionally, the DOI for each article was added whenever available. The authors have also checked that links for online references and all links were accessible. The authors did not note any retracted references. |
| Reviewer’s comments |  |
| This paper just need few revisions Please, change online- learning to remote education | The change was made in the title and throughout the paper when applicable. |
| Could you give more information regarding the setting where the data was collected  More information regarding informed consent and how you maintain the research ethics is required | More information was added about the setting within the methodology. The setting reflected that of the online environment where the interviews took place.  The following section was added “Interviews were conducted online using Microsoft Teams (MS Teams). MS Teams is a known video conferencing platform which was commonly used among Qatar University students and staff. Due to the COVID-19 pandemic participants and researchers resorted to conducting the interviews from their home setting. To comply with ethical requirements, participants were asked to place themselves in quiet room with minimal distraction within their home. Similarly, researchers used a room with minimal distraction and ensured the use of headphones to maintain confidentiality.”  Additionally, more information was added under the Ethics section. “Once a participant is identified to meet the inclusion criteria, they were emailed a participant information sheet which included information about the study and a consent form, seven days prior to the interview. Participants were directed to contract the research term if they have any concern or questions during this period. If they agree to participate, they were required to sign the consent form and send it to the principal researcher (MJ). Additionally, before the start of the interview, the participants were reminded of the study objective and confirmation to participate was also obtained. Interview audio recordings were saved for transcribing purposes only after which they were permanently deleted. Consent forms and transcribed data were all kept in Qatar University password protected device in compliance with Qatar University Institutional Review Board (QU-IRB).” |
| Could you explain more regarding recruitment and data collection More information is required regarding inclusion criteria for the participants Take a look for a study in the area such as Abuhammad S. Barriers to distance learning during the COVID-19 outbreak: A qualitative review from parents’ perspective. Heliyon. 2020 Nov 10:e05482 | More information was added into the manuscript “Participants were identified through the research team and through communication with the respective department heads.” and “Inclusion criteria included faculty members teaching within the Health Cluster who were involved with the design and/or delivery of remote teaching sessions with students. Students were eligible if they were enrolled within the colleges of the Health Cluster and have received remote education as part of their university courses. Exclusion criteria included any participant who meets the inclusion criteria but did not provide consent.”  The study suggested by the reviewer reflects on the parents perceptions and barriers to remote education. The reference was reviewed and found to add benefit within the discussion. |
